# The efficacy of antihypertensive drugs in chronic intermittent hypoxia conditions

**DOI:** 10.3389/fphys.2014.00361

**Published:** 2014-09-22

**Authors:** Lucilia N. Diogo, Emília C. Monteiro

**Affiliations:** Centro de Estudos de Doenças Crónicas, CEDOC, NOVA Medical School/Faculdade de Ciências Médicas, Universidade Nova de LisboaLisboa, Portugal

**Keywords:** antihypertensive drugs, blood pressure, chronic intermittent hypoxia, hypertension, obstructive sleep apnea

## Abstract

Sleep apnea/hypopnea disorders include centrally originated diseases and obstructive sleep apnea (OSA). This last condition is renowned as a frequent secondary cause of hypertension (HT). The mechanisms involved in the pathogenesis of HT can be summarized in relation to two main pathways: sympathetic nervous system stimulation mediated mainly by activation of carotid body (CB) chemoreflexes and/or asphyxia, and, by no means the least important, the systemic effects of chronic intermittent hypoxia (CIH). The use of animal models has revealed that CIH is the critical stimulus underlying sympathetic activity and hypertension, and that this effect requires the presence of functional arterial chemoreceptors, which are hyperactive in CIH. These models of CIH mimic the HT observed in humans and allow the study of CIH independently without the mechanical obstruction component. The effect of continuous positive airway pressure (CPAP), the gold standard treatment for OSA patients, to reduce blood pressure seems to be modest and concomitant antihypertensive therapy is still required. We focus this review on the efficacy of pharmacological interventions to revert HT associated with CIH conditions in both animal models and humans. First, we explore the experimental animal models, developed to mimic HT related to CIH, which have been used to investigate the effect of antihypertensive drugs (AHDs). Second, we review what is known about drug efficacy to reverse HT induced by CIH in animals. Moreover, findings in humans with OSA are cited to demonstrate the lack of strong evidence for the establishment of a first-line antihypertensive regimen for these patients. Indeed, specific therapeutic guidelines for the pharmacological treatment of HT in these patients are still lacking. Finally, we discuss the future perspectives concerning the non-pharmacological and pharmacological management of this particular type of HT.

## Chronic intermittent hypoxia-related disorders

Is is well established that intermittent hypoxia (IH) affects control of breathing, the autonomic nervous system and the cardiovascular system (Foster et al., 2007). Chronic intermittent hypoxia (CIH) is a feature that is present in interstitial lung disease (Fletcher et al., 1992a) and sleep-disordered breathing (SDB), and it has also been shown to occur in patients with hepatopulmonary syndrome (Tanné et al., 2005; Ogata et al., 2006; Palma et al., 2008). Since several years ago, there has been growing interest concerning CIH due to the high relevance of the part assumed to be played by sleep-related breathing disorders in chronic diseases.

Sleep apnea/hypopnea disorders include centrally originated diseases and obstructive sleep apnea (OSA). Central sleep apnea (CSA) is characterized by a lack of drive to breathe during sleep, resulting in insufficient or absent ventilation and compromised gas exchange (Eckert et al., 2007). In CSA, the cessation of respiration during sleep is not associated with ventilatory effort and there is sleep fragmentation due to arousals associated with reflexes activated by the ensuing hypoxemia (Paiva and Attarian, 2014). The major manifestations of CSA include high altitude-induced periodic breathing, idiopathic CSA, narcotic-induced central apnea, obesity hypoventilation syndrome, and Cheyne-Stokes breathing in heart failure (Eckert et al., 2007). While the precipitating mechanisms involved in the several types of CSA may diverge, unstable ventilatory drive during sleep is the principal underlying feature (Eckert et al., 2007). CSA is diagnosed in approximately 5% of the patients who undergo a polysomnographic study (Khan and Franco, 2014). On the other hand, OSA is briefly characterized by repetitive episodes of airflow cessation (apnea) or airflow reduction (hypopnea) caused by an obstructed or collapsed upper airway during sleep. Unlike CSA, obstruction occurs in OSA despite the central drive to breathe and inspiratory muscle activity (Levitzky, 2008). An appreciable number of factors are known to be linked to upper-airway collapse, namely reduced airway dilator muscle activity during sleep, upper-airway anatomy, obesity, decreased end-expiratory lung volume, ventilatory control instability, and rostral fluid shifts (Kapur, 2010). The repetitive episodes of apnea and hypopnea characteristic of OSA are closely associated with CIH, hypercapnia and an increase in intrathoracic pressure, leading to recurrent arousals and significant changes in sleep architecture. OSA is affecting a growing proportion of the common population, and the estimated prevalence in the 1990s was 9% for women and 24% for men among middle-aged adults (Young et al., 1993). In addition, CSA can occur concomitantly with OSA. This last condition, recently labeled complex sleep syndrome, is observed in approximately 15% of the patients following treatment with continuous positive airway pressure (CPAP) (Paiva and Attarian, 2014). In a few words, complex sleep syndrome is a form of SDB in which CSA persists or emerges when obstructive events have disappeared using a positive pressure device (Khan and Franco, 2014). In clinical practice, when a few central apneas are observed in polysomnograms of patients with OSA, they are normally ignored because we do not presently understand their potencial clinical relevance.

Nowadays, it is well known that the outcomes of these sleep-related breathing disorders can lead to vascular diseases, contributing to a considerable increase in overall cardiovascular risk. The desaturation-reoxygenation sequence, a typical pattern coupled with the majority of respiratory events, is thought to be responsible for most of the associated cardiovascular morbidity (Lévy et al., 2012). Although OSA has been associated with several cardiovascular conditions, it has been more closely etiologically connected to systemic HT (Kapa et al., 2008), and the link between HT and OSA is now widely accepted and supported by different findings. Most episodes of OSA are coupled with sleep disruption, which *per se* increases sympathetic nerve activity and blood pressure (Morgan et al., 1996). In addition, the occurrence of arousals appears to enhance the pressor effects of asphyxia during OSA (Morgan et al., 1998), contributing synergistically to blood pressure increase. In any case, studies in both animals and humans underline the major role of hypoxia itself in promoting an increase in blood pressure (Brooks et al., 1997b; Tamisier et al., 2011).

Regarding CSA, this SDB, like OSA, is strongly linked to cardiac disease and cardiovascular outcomes (Brenner et al., 2008). Indeed, the majority of patients with CSA have underlying cardiovascular disease, primarily heart failure, which is considered the most common risk factor for CSA, followed by atrial fibrillation (Bradley and Phillipson, 1992). Moreover, like OSA, CSA has been implicated in heart failure pathophysiology (Mehra, 2014) and occurs in 30–50% of patients with left ventricular dysfunction and heart failure caused by HT, cardiomyopathy and ischemic heart disease (Bradley and Floras, 2003). Thus, CSA has significant co-morbidity with many cardiac conditions, which clearly contributes to an increase in the associated mortality and morbidity.

Besides systemic HT, chronic intermittent alveolar and systemic arterial hypoxia-hypercapnia can cause pulmonary HT (PH). SDB has also been found to be associated with PH, being considered one of the potential etiologies of PH (Galie et al., 2009). During episodes of OSA, the subsequent oscillations in PaO_2_ lead to a cyclical pattern of vasoconstrictions and relaxations in the pulmonary circulation responsible for the marked fluctuations observed in pulmonary arterial pressure (Dempsey et al., 2010). The perpetuation of this pattern leads to fixed elevations in pulmonary pressure (Dempsey et al., 2010). Some data suggest that even slight changes in pulmonary function, in the absence of lung disease, are able to induce PH in patients with OSA. Furthermore, it is important to bear in mind that PH could also be a cause of abnormal arterial blood gases during wakefulness (Dempsey et al., 2010) and that OSA itself can lead to PH (Sajkov and McEvoy, 2009). The major consequence of the increased pulmonary artery pressure, together with increased blood viscosity (a consequence of the renal release of erythropoietin subsequent to hypoxemia), is the occurrence of right ventricle hypertrophy leading to *cor pulmonale* (Levitzky, 2008). The prevalence of this chronic cardiopulmonary condition among patients with SDB is estimated to range from 17 to 52% (Minic et al., 2014), and 20–30% of untreated OSA patients suffer from PH (Dumitrascu et al., 2013). Even if PH in this group of patients is typically not severe (Badesch et al., 2010), OSA patients with PH have a higher mortality rate than OSA patients without PH (Minai et al., 2009). A recent meta-analysis shows that CPAP is associated with a mild but statistically significant reduction in pulmonary artery pressure in OSA patients (Sun et al., 2014). This decrease might translate into a better outcome in patients with PH secondary to OSA. However, more studies are needed to confirm this assumption.

Taking into account its high prevalence and its associated adverse impact on cardiovascular, metabolic and other health outcomes, this review focuses on OSA and systemic HT.

## OSA and HT: how relevant is this linkage?

Since 2003, OSA has formally been recognized as a frequent and important secondary cause of HT and is one of the first causes to be screened mainly in patients with a suggestive phenotype, refractory HT and a non-dipping profile (Chobanian et al., 2003; Mancia et al., 2007). More recently, OSA has been identified as an independent risk factor for HT (Lavie et al., 2000; Peppard et al., 2000; Marin et al., 2012), as one of the major clinical conditions that favors poorly controlled HT (Oliveras and Schmieder, 2013), and as the most common condition associated with resistant HT (Pedrosa et al., 2011). OSA and HT are two prevailing risk factors for several cardiovascular events (Wang and Vasan, 2005; Baguet et al., 2009). Due to their high prevalence and cardiovascular morbidity (Wolf et al., 2007; Malhotra and Loscalzo, 2009), OSA and HT are now acknowledged as public health problems. Epidemiological data show that the estimated overall prevalence of HT among patients with OSA is approximately 50% and an estimated 30–40% of hypertensive patients are diagnosed with OSA (Calhoun, 2010), confirming the bidirectional relationship between OSA and HT. Moreover, OSA and HT are chronic diseases mostly diagnosed in active adults and because of the associations between OSA and obesity and advancing age, the public health burden of OSA related to cardiovascular disease is expected to rise in the coming years (Dempsey et al., 2010). The use of both antihypertensive drugs (AHDs) and CPAP in these patients is for life and consequently treatment is associated with a high impact both in terms of costs and in patients' quality of life. Indeed, OSA generates an impressive economic burden, including medical costs, when compared to other equally relevant chronic diseases (Kapur, 2010; Badran et al., 2014).

## OSA and HT: what is the problem?

CPAP is considered the gold standard treatment for mild, moderate and severe OSA due to its remarkable ability in providing pneumatic splitting of the upper airway and effectiveness in reducing the apnea-hypopnea index (AHI), symptoms, and cardiovascular morbidity and mortality (Hla et al., 2002; Pepperell et al., 2002; Wolf et al., 2007; Epstein et al., 2009; Mannarino et al., 2012). Besides preventing hypoxemia, sleep disturbance and apnea episodes, CPAP reduces sympathetic activity, systemic inflammation and oxidative stress (Yorgun et al., 2014). However, the results found for the effectiveness of CPAP on blood pressure (BP) control are still controversial. Table 1 summarizes the results of original studies in which the effect of CPAP on BP has been analyzed. Whereas some studies and meta-analyses (Bakker et al., 2014; Varounis et al., 2014) have reported modest effects for CPAP in lowering BP, others tend to support the beneficial effect of CPAP treatment on BP reduction and attenuating the risk of developing HT. In any case, although the lowering effect of CPAP on BP is relevant in terms of overall cardiovascular risk reduction, this effect is very limited when compared to the performance of AHDs in patients with essential HT (Pépin et al., 2009). Thus, treating HT in patients with sleep apnea is proving to be a difficult task and there is consensus that the use of AHDs is mandatory. In spite of this, data on AHDs regimens in patients with OSA are scarce and there is a lack of specific therapeutic guidelines for the pharmacological treatment of HT in these patients. Furthermore, the effects of AH agents on OSA patients are not consistent (Parati et al., 2012) and there are no data on the efficacy of specific AHDs regimens when associated with CPAP.

**Table 1:** **CPAP effect on blood pressure**.

**Study design**	**n**	**Study duration**	**HT patients (%)**	**AHDs (Y/N)**	**Mean CPAP use (h/night)**	**BP outcome**	**References**
RCT; parallel group; blinded endpoint	194	12 weeks	100	Yes	5	↓ 3.1 mmHg MBP ↓ 3.2 mmHg DBP ↓ 3.1 mmHg SBP (NS)	Martínez-García et al., 2013
RCT; parallel group	118	4 weeks	10	Yes	4.9	↓ 3.3 mmHg 24 h MBP	Pepperell et al., 2002
Case -controlled study	48	4 weeks	79	Yes	5.1	↓ 5.2 mmHg DBP ↓ 3.8 mmHg SBP (NS)	Zhao et al., 2012
Prospective randomized trial	32	9 weeks	66	Yes	5.5	↓ ± 10 mmHg MBP ↓ ± 10 mmHg DBP ↓ ± 10 mmHg SBP (During both day and night-time)	Becker et al., 2003
RCT; multicenter; parallel group	723	4 years	51.5	Yes	5.0	NS on new-onset HT	Barbé et al., 2012
Prospective, single-center, long-term follow-up	91	5 years	100	Yes	*NA*	NS on 24 h BP, SBP and DBP	Kasiakogias et al., 2013
RCT; parallel group	40	6 months	100	Yes	6.01	↓ Awake SBP (6.5 mmHg) and DBP (4.5 mmHg) NS nocturnal SBP and DBP	Pedrosa et al., 2013
Retrospective chart review study	98	1 year	100	Yes	6.3	↓ 5.6 mmHg MBP (resistant HT group) ↓ 0.8 mmHg MBP (controlled BP group)	Dernaika et al., 2009
RCT; crossover	28	8 weeks	100	Yes	4.8	↓ 2.1 mmHg 24 h MBP (CPAP group) ↓ 9.1 mmHg 24 h MBP (valsartan group)	Pépin et al., 2009
Prospective cohort study	86	6 months	55	Yes	4.8	↓ 4.92 mmHg 24 h MBP	Robinson et al., 2008
Observational study	24	12 weeks	0	No	*NA*	↓ 5.3 mmHg 24 h MBP	Yorgun et al., 2014
Prospective cohort study	196	6 months	85	Yes	*NA*	↓ 2.7 mmHg DBP ↓ 2.1 mmHg SBP	Börgel et al., 2004
RCT; multicenter; double-blinded	340	12 weeks	100	No	4.5	↓ 1.5 mmHg MBP ↓ 1.3 mmHg mean DBP ↓ 2.1 mmHg mean SBP	Durán-Cantolla et al., 2010
RCT; multicenter; parallel group	44	6 weeks	*NA*	Yes	5.0	NS on 24 h SBP and DBP	Barbé et al., 2001
RCT; crossover study; sham placebo	35	10 weeks	100	Yes	5.2	NS on overall 24 h MBP	Robinson et al., 2006
Observational, monocentric; cohort study	495	3.4 years	40.4	Yes	*NA*	↓ Occurrence of systemic arterial HT	Bottini et al., 2012
RCT; single-blinded	44	13.2 weeks	100	Yes	5.1	Additional ↓ in office BP and ambulatory BP monitoring (CPAP+ 3 AHDs)	Litvin et al., 2013
RCT, multicenter	359	1 year	100	Yes	4.7	↓ 2.19 mmHg DBP NS ↓ 1.89 mmHg SBP NS	Barbé et al., 2009
RCT	36	3 months	*NA*	No	5.2	↓ 2 mmHg office DBP ↓ 5 mmHg office SBP ↓ 5 mmHg 24 h DBP ↓ 5 mmHg 24 h SBP	Drager et al., 2011
RCT; parallel group	64	3 months	100	Yes	>5.8	↓ 6.98 mmHg 24 h DBP ↓ 9.71 mmHg 24 h SBP	Lozano et al., 2010

*AHDs, antihypertensive drugs; BP, blood pressure; CPAP, continuous positive airway pressure; DBP, diastolic blood pressure; HT (%), percentage of hypertensive patients; MBP, mean blood pressure; NA, information not available; NS, no significant effect; RCT, randomized controlled trials; SBP, systolic blood pressure; ↓, decrease*.

A new treatment for OSA patients is the oral appliance/mandibular advancement device (Guralnick and Bakris, 2012). Oral appliance therapy is an important alternative to CPAP for some patients with mild to moderate OSA (Iftikhar et al., 2013). Despite a recent study (Andrén et al., 2013) and a recent meta-analysis (Iftikhar et al., 2013) which have shown some beneficial effects of this device in reducing blood pressure measurements, larger and longer randomized control trials are needed to confirm the effects of oral appliance therapy on BP control.

Clearly, more studies are required to identify first-line AHDs regimens for optimal BP control in this particular group of hypertensive patients (Tsioufis et al., 2010; Parati et al., 2013). Moreover, HT related to OSA needs to be managed as a specific entity and an earlier diagnosis of this type of HT seems to be as relevant as the selection of AHDs regimens. This work provides, for the first time, a systematic review on the efficacy of AHDs in HT related to OSA.

## What models are available to study HT related to OSA?

Due to the high complexity and heterogeneity associated with OSA, considerable variability can be observed between reports addressed at the study of this disease. In addition, the scarcity of opportunities for patient investigation, in particular at the cellular level, has compromised progress in understanding the pathophysiology of OSA and the development of novel and specific treatments for this disorder. To overcome some of these limitations, several animal models and more recently, a model of OSA in healthy human volunteers (Tamisier et al., 2009, 2011) have been developed. Animal models, especially of IH, mimic OSA more easily than human models. The small size of rodents allows more rapid and intense changes in SaO_2_ whereas humans require longer periods of hypoxia to induce arterial oxyhaemoglobin desaturation (Foster et al., 2007). The combination of these two approaches is certain to contribute to the consolidation of prevention strategies and the development of more suitable treatments for OSA patients.

### Animal models

The major advantage of the use of animal models is that they allow single components of the disease to be evaluated, accurately controlling the triggering events in terms of both severity and duration, and providing homogeneous populations (Lévy et al., 2012). These models also provide an excellent opportunity to explore the underlying mechanistic pathways of HT related to OSA and their consequences under controlled conditions. Moreover, animal models have enabled the study of parameters that have proved difficult to assess in humans, particularly due to the need for organ harvesting to explore the mechanisms underlying the consequences of IH at the molecular level (Dematteis et al., 2009). Thus, studies with animal models are good tools for overcoming some confounding factors present in human studies (e.g., the presence of comorbidities, disease duration, and behavioral and environmental variables) (Badran et al., 2014), and for providing more specific information concerning the efficacy of drugs to be tested.

In 2009, Dematteis et al. used the terminology homologous (sharing the cause or pathophysiology of the human disease), predictive (responding to treatment similarly to the human disease) and isomorphic (displaying symptoms similar to those of the human disease although their cause and pathophysiology may differ) to categorize sleep apnea models (Dematteis et al., 2009). According to these categories, most sleep apnea models are only partially isomorphic, focusing on a specific aspect of the human disease. As a matter of fact, none of the currently available animal models reproduce all aspects of human sleep apnea and they present some important limitations. Nonetheless, the animal models of sleep apnea have brought out most of the available knowledge in this field and furthermore, almost all cardiovascular diseases known to be present in patients with OSA have been replicated in these models (Dumitrascu et al., 2013).

The effective use of animals to study sleep apnea implies recognition of the natural similarities and differences between animals and humans to ensure the reliability of the experimental results. For instance, as rodents are nocturnal animals, the stimulus must be applied during the sleep-dominant phase of the diurnal cycle. Moreover, in humans the circadian distribution of sleep tends to be consolidated and normally monophasic, with a daily sleep duration of 7–8 h, whereas it is polyphasic, relatively fragmented and with a duration of 12–15 h in rodents (Toth and Bhargava, 2013). Another issue is related to the fact that rodents sleep in the prone position (Golbidi et al., 2012); it is well known that supine OSA is the dominant phenotype of OSA syndrome and that the supine position favors upper airway collapse in humans (Joosten et al., 2014). Furthermore, additional care must be taken to minimize external factors (e.g., light exposure, photoperiod, noise, disruptions in the home environment, and post-surgical care in studies, for instance requiring implantation of telemetric devices) able to influence sleep in animals used in experimental research (reviewed in Toth and Bhargava, 2013).

The experimental animal models developed to mimic OSA have recently been reviewed (Dematteis et al., 2009; Golbidi et al., 2012; Davis and O'Donnell, 2013; Toth and Bhargava, 2013) and assembled taking into account the main injuries triggered by OSA. Despite attempts to use large animals (e.g., dogs, lambs, and pigs) to simulate upper airway obstruction, most research on the cardiovascular consequences of OSA has been performed in rodents. Alternative models (e.g., cell cultures incubated in specific devices that perform oxygen fluctuations mimicking sleep apnea-related IH), mainly relevant to signaling investigation (Kumar et al., 2003; Gozal et al., 2005; Ryan et al., 2005), represent a complementary approach to the most widely used sleep models. However, in spite of the recommendations to refine, reduce and replace (the 3Rs programme), these alternative models cannot replace animal models in the study of HT.

The natural models of sleep apnea include the English bulldog, the historic natural model of spontaneous obstruction (Hendricks et al., 1987), the sleep-related central apnea models [e.g., Sprague-Dawley rats (Carley et al., 2000), spontaneously hypertensive (SH) rats (Carley et al., 1998), C57BL/6J (Julien et al., 2003; Liu et al., 2010)], and the Zucker obese rat in which apnea is obesity-related (Ray et al., 2007; Lee et al., 2008; Iwasaki et al., 2012). The experimentally-induced models (e.g., the sleep deprivation model, induced airway obstruction and the CIH model) are the most widely used. Due to model limitations and lack of extensive study, we only briefly describe the induced airway obstruction model and the sleep deprivation model. Special focus will be given to the CIH model, based on the assumption that IH is the most effective paradigm to induce HT related to OSA and probably the most relevant stimulus regarding the cardiovascular sequelae of OSA.

#### Induced airway obstruction model

Briefly, the airway obstruction model involves surgical intervention (an endotracheal tube), which is an invasive procedure, or alternatively the use of a specific chamber with a latex neck collar that induces recurrent airway obstruction. This latter procedure, developed by Farré et al. (2007), is associated with high levels of stress due to the restriction of animal movement. In both approaches, the degree of obstruction is adjustable (Golbidi et al., 2012) and in the case of induction of obstruction through endotracheal tube, the PaCO_2_ can be adjusted to mimic human sleep apnea (Golbidi et al., 2012). Many experiments using this method have not monitored the sleep state of the animals, but more recent studies have incorporated sophisticated apparatus that is able to detect sleep-awake states and allow close coordination between the initiation of airway obstruction and sleep onset (Schneider et al., 2000).

This model allows the study of the potential consequences of strenuous breathing against an obstructed airway and can be used to study the cardiovascular consequences and risk factors of OSA (e.g., systemic inflammation and coagulation), and to investigate the mechanisms that underlie OSA (Salejee et al., 1993; Nácher et al., 2007, 2009; Almendros et al., 2008, 2011; Othman et al., 2010). However, to the best of our knowledge, no study has yet shown that this obstruction model is able to mimic HT related to OSA. Furthermore, when testing AHDs, it became crucial to ensure the selection of a stress-free paradigm as it has been shown that any source of external stress on rodents can significantly increase heart rate and blood pressure (Brown et al., 2000; Kramer et al., 2000; Balcombe et al., 2004; Bonnichsen et al., 2005) and therefore contribute to confounding the experimental results. Finally, as the rat models of obstruction or asphyxia were developed in restrained or anesthetized rats, they are not good models for chronic administration of oral drugs, particularly AHDs.

#### Sleep deprivation model

In the last few years, several approaches have been used to trigger sleep deprivation in different animals, the rat being the animal of choice to date (Colavito et al., 2013). In the “multiple platform technique,” the animal is aroused from sleep when the characteristic loss of muscle tone that accompanies paradoxical sleep causes it to fall off the platform (Suchecki and Tufik, 2000). The “gentle handling” procedure, by far the most popular method, is based on direct interaction with the experimenter, who actively keeps the animal awake through the use of external stimulation (e.g., mild noises, tapping or gentle shaking of the cage, or by direct contact with the animal either using a soft brush or by hand), or by the introduction of novel objects or nesting material in the cages, which typically leads to active exploratory behavior (Colavito et al., 2013).

These models are most often used to evaluate the neurophysiological aspects of OSA (Van Dongen et al., 2003; Haack and Mullington, 2005; McKenna et al., 2007; Ward et al., 2009; Nair et al., 2011) due to the high similarity between the structures of the nervous systems of rodents and humans (Badran et al., 2014), and to illustrate some mechanistic pathways induced by this trigger (McGuire et al., 2008; Tartar et al., 2010; Liu et al., 2011; Perry et al., 2011). Nevertheless, some studies have also aimed to evaluate the cardiovascular outcomes induced by this OSA feature and have suggested that sleep fragmentation may have a far more important role in cardiovascular changes observed in OSA patients (Golbidi et al., 2012). Even so, sleep deprivation studies have produced mixed results regarding BP outcomes.

In 1997, Brooks et al. suggested that sleep fragmentation, triggered by auditory stimulus, induced only acute changes in BP and did not affect daytime BP (Brooks et al., 1997a,b). In the same way, Bao et al.'s results showed that sleep fragmentation in rats, using acoustic stimuli for 35 days, did not elicit an increase in BP, probably due to some adaptation behavior (Bao et al., 1997). However, more recent studies have shown that sleep deprivation leads to increased plasma concentrations of epinephrine and norepinephrine (Andersen et al., 2005), ET-1/2 levels (Palma et al., 2002), and increased heart rate and systolic blood pressure (Andersen et al., 2004; Perry et al., 2007). In addition, sleep fragmentation enhances plasma inflammatory cytokines (e.g., TNF-α, IL-6, IL-1α, and IL-1β), leading to increased oxidative stress and inflammation (Yehuda et al., 2009). These results add further evidence demonstrating that sleep deprivation may lead to serious cardiovascular consequences and may aggravate hypertensive features. However, despite the potential of the sleep deprivation model to induce HT related to OSA, it does not exactly mimic sleep fragmentation and presents one major shortcoming regarding the evaluation of AHDs efficacy that should be taken into account. Sleep deprivation is a stressful method and it is still unclear whether the method is itself a stressful stimulus (Palma et al., 2002). Thus, in conclusion, sleep deprivation models are useful tools for unveiling various aspects of sleep function, studying the effects of sleep loss on subsequent brain function at the molecular, cellular and physiological levels, and evaluating cognitive impairment, but should be used with caution whenever stress can act as a confounding factor and compromise data interpretation.

#### CIH model

IH is now established as the dominant model of sleep apnea. Generally, this model makes use of specific ventilated chambers in which the animals are housed and cyclically exposed either to normoxia/hypoxia or room air to mimic the most relevant consequences of OSA. Hypoxic conditions can also be achieved by surgical intervention (an endotracheal tube) or by the use of a mask, which involves animal restraint and consequently high levels of stress (Golbidi et al., 2012). In either case, animals breathe nitrogen-enriched air alternating with oxygen or normal air (Dematteis et al., 2009). Thus, as with O_2_, nitrogen plays an important role in this model as the flushing of the chambers with this gas allows the gradual lowering of O_2_. The duration of the hypoxic and normoxic phases of the IH cycle, as well as the slopes of FiO_2_, decrease and increase, and are dependent on cage/chamber size and the gas flows and mixtures (Dematteis et al., 2009).

The standard animal model of OSA was that described in the landmark study of Fletcher and Bao (1996). Despite the presence of some drawbacks, this model has successfully been employed to study the changes in systemic arterial pressure and the impact of IH on a wide range of cardiovascular outcomes. One of the major limitations pointed to in this model is the absence of recurrent upper airway obstruction, abolishing the acute hemodynamic changes due to the negative intrathoracic pressure (Badran et al., 2014). Marked negative intrathoracic pressure induces acute hemodynamic changes that are probably the starting point for chronic cardiovascular diseases (Bonsignore et al., 1994). Despite the absence of upper airway occlusion, some respiratory efforts (intermittent tachypnea) occur, corresponding to a fluctuating hyperventilation that follows the IH cycles (Dematteis et al., 2009). However, this disadvantage allows the evaluation of CIH effects, namely chronic blood gas exchanges, without the interference of the mechanical aspects of OSA.

This model also fails to reproduce the transient hypercapnia, or at least eucapnia, which occurs in humans determined by airway occlusion. The first question concerning this issue should be: is PaCO_2_ relevant in humans? Hypercapnia is not a standard parameter analyzed in polysomnographic recordings in patients and therefore there is no consensus on the impact of PaCO_2_ in arterial blood pressure in patients with OSA. In clinical studies of patients with moderate OSA, the changes in PaCO_2_ have seemed to be irrelevant (Epstein et al., 2001) or have shown a slight increase (Tilkian et al., 1976) during the apneic events. However, a PaCO_2_ increase may contribute to the severity of the cardiovascular consequences of OSA (Cooper et al., 2005). The results shown by Fletcher et al. in rats suggest that the exposure to hypercapnia during IH is not a critical factor as the effect of IH on diurnal BP is similar, independently of the lower or higher levels of CO_2_ (Fletcher et al., 1995). Moreover, Bao et al. found that eucapnic IH in rats is a more powerful stimulus for inducing acute BP increase than hypocapnic IH (Bao et al., 1997). Similarly, Lesske et al. showed comparable changes in BP between two groups submitted to IH with or without hypercapnia (Lesske et al., 1997). On the other hand, based on the results of different CIH experimental protocols in rodents, Kanagy concludes that the level of PaCO_2_ influences the magnitude of an increase in BP (Kanagy, 2009). Concretely, eucapnic hypoxia induces a faster and greater increase than hypocapnic hypoxia (Kanagy, 2009), through mechanisms that presently remain unknown. Moreover, the greatest increases in BP have been observed in studies in which hypocapnia was prevented by CO_2_ administration (Morgan, 2009). Likewise, Tamisier et al., in a study performed in humans, reported that hypercapnic hypoxia leads to greater sympathetic activation than hypocapnic hypoxia (Tamisier et al., 2009). In line with these findings, the presence of hypocapnic or eucapnic hypoxia conditions leads to an underestimated increase in BP that must be taken into account. In conclusion, although some data suggest that PaCO_2_ may influence physiological responses to IH, further studies are needed to evaluate the combined effect of IH and hypercapnia. Another drawback that could be attributed to the IH paradigm is the fact that it is not accompanied by sleep fragmentation and does not incorporate monitoring of sleep.

Each group of researchers has applied its own specific paradigm and these discrepancies may compromise the straightforward comparison of the results. The several paradigms of CIH, which simulate the cyclical pattern of hypoxia experienced by patients with OSA, diverge in some respects, namely in the animal species involved, e.g., Sprague-Dawley rats (Fletcher et al., 1995; Kanagy et al., 2001; Tahawi et al., 2001; Allahdadi et al., 2005; Chen et al., 2005; Phillips et al., 2005; Lai et al., 2006), Wistar rats (Dunleavy et al., 2005; Lefebvre et al., 2006), C57BL/6J mice (Julien et al., 2003), and CF-1 mice (Rosa et al., 2011), the severity of hypoxia, the number of hypoxic episodes *per* hour of sleep, the number of days of hypoxic exposure (exposure duration), and CO_2_ manipulation. Table 2 summarizes the variability observed in the CIH models.

**Table 2:** **Reports on the effects of CIH on blood pressure**.

**Species**	**Hypoxia cycle, Nadir FiO_2_, duration and CO_2_ manipulation (Y/N)**	**BP measurement**	**Effect on BP**	**References**
Sprague-Dawley rats	20 cycles (90 s each) of 21–5% O_2_ and 0–5% CO_2_/h; 7 h/day; 35 days; Yes	Tail-cuff method	↑ MAP (25–28 mmHg)	Allahdadi et al., 2005
Sprague-Dawley rats	80 cycles (6 min each) 21–10% O_2_/day; 8 h/day; 7 days; No	Telemetry	↑ MAP (7–10 mmHg)	Knight et al., 2011
Sprague-Dawley rats	5% O_2_ 12 times/h; 8 h/day; 7–21 days; No	Arterial catheterization	No changes in MAP	Iturriaga et al., 2010
Wistar rats	10% O_2_ for 4 h/day and 21% O_2_ for 20 h/day; 56 days; Yes (PCO_2_ < 0.02%)	Arterial catheterization	No differences in systemic pressure	Kalaria et al., 2004
Sprague-Dawley rats	2/3–20.9% O_2_ (3–6 s +15–18 s; 2 cycles/min); 6–8 h/day; 35 days; No	Telemetry	↑ MAP (16 mmHg)	Tahawi et al., 2001
C57BL/6J mice	21–5% O_2_ (60 s); 12 h/day; 5 weeks; No	Arterial catheterization	↑ Systemic BP (7.5 mmHg)	Campen et al., 2005
SHR + Wistar rats	21–10% O_2_ (1 min cycles: 20 s + 40 s); 8 h/day; 14 days; No	Tail-cuff method + Arterial catheterization	Enhanced HT development in SHR + NS in Wistar rats	Belaidi et al., 2009
Sprague-Dawley rats	2/3–20.9% O_2_ (3–6 s + 12 s; 2 cycles/min); 6–8 h/day; 35 days; No	Telemetry	↑ MAP (10 mmHg)	Fletcher, 2000
LCR and HCR	21–10% O_2_ (3 min cycles); 8 h/day; 7 days; No	Telemetry	↑ MAP in both groups	Sharpe et al., 2013
Sprague-Dawley rats	20 cycles (90 s each) of 21–5% O_2_ and 0–5% CO_2_/h; 8 h/day; 11 days; Yes	Arterial catheterization	↑ MAP (30 mmHg)	Kanagy et al., 2001
Sprague-Dawley rats	48 cycles (45 + 30 s) 20.9–2/6% O_2_/h; 6 h/day; 30 days; No	Telemetry	↑ MAP (19.3 mmHg)	Lai et al., 2006
C57BL/6J mice	21–5.7% O_2_ (alternating every 6 min); 12 h/day; 90 days; No	Arterial catheterization	↑ MAP (19.8 mmHg)	Lin et al., 2007
Sprague-Dawley rats	21–10% O_2_ for 5 s every 90 s; 10 h/day; 4 weeks; No	Tail BP telemeter	↑ MAP (37 mmHg)	Liu et al., 2013
SHR	21–10% O_2_ (alternating every 90 s); 12 h/day; 30 days; No	Tail-cuff method	↑ SBP and DBP (NA mmHg)	Soukhova-O'Hare et al., 2008
Sprague-Dawley rats	21–4/6% O_2_ (every 60 s); 8 h/day; 5 days/week; 5 weeks; No	Tail-cuff method	↑ MAP (12 mmHg)	Chen et al., 2005
Wistar rats	1 min cycles with 30 s of a 5% FiO_2_; 8 h/day; 14–21 days; No	Tail-cuff method + Arterial catheterization	Rapidly ↑ MAP (NA mmHg)	Totoson et al., 2013
Sprague-Dawley rats	21–6% O_2_ (9 min cycles); 8 h/day; 14 days; No	Arterial catheterization	↑ MAP (9 mmHg)	Silva and Schreihofer, 2011
Wistar rats	20.8–6% O_2_ (9 min cycles: 5 min Nx); 8 h/day; 10 days; No	Arterial catheterization	↑ MAP (12 mmHg) ↑ SBP (9 mmHg) ↑ DBP (8 mmHg)	Zoccal et al., 2009
Wistar- Hannover rats	21–10% O_2_ (2 min + 2 min); 1000–1600 h; Yes (PCO_2_ < 0.01%)	Arterial catheterization	No differences in MAP	Perry et al., 2011
Sprague-Dawley rats	10 cycles (6 min each) of 21–6% O_2_ and 0–5% CO_2_/h; 8 h/day; 28 days; Yes	Telemetry	↑ SBP (39 mmHg) ↑ DBP (33 mmHg)	Dyavanapalli et al., 2014
C57BL/6J mice	21–7% O_2_ (120 s each cycle); 5 days/week; 8 h/day; 6 weeks; No	Telemetry	Significant ↑ MAP	Schulz et al., 2014
Sprague-Dawley rats	21–10% O_2_ (cycle duration: NA); 8 h/day; 7 days; No	Telemetry	↑ MAP that persisted after CIH exposure	Bathina et al., 2013

*BP, blood pressure; CIH, chronic intermittent hypoxia; DBP, diastolic blood pressure; h, hour; HCR, high aerobic capacity rats; HT, hypertension; LCR, low aerobic capacity rats; MAP, mean arterial pressure; NA, information not available; NS, no significant effect; Nx, normoxia; min, minutes; s, seconds; SBP, systolic blood pressure; SHR, spontaneously hypertensive rats; ↑, increase*.

These models typically create moderate to severe oxygen desaturation, thereby mimicking severe forms of OSA and may therefore not be applicable to mild and moderate clinical OSA (Dematteis et al., 2009). CIH models with cycles of FiO_2_ of 5% or less usually mimic severe forms of OSA in humans and produce maximal changes in BP and heart rate (Dematteis et al., 2008). However, higher FiO_2_ (8–10%) has been used in rodent models of CIH (Soukhova-O'Hare et al., 2008; Knight et al., 2011; Perry et al., 2011; Bathina et al., 2013).

The duration and frequency of hypoxic/normoxic periods are adjustable; usually, the higher the frequency the shorter the IH cycles (Golbidi et al., 2012). There is a sizeable discrepancy regarding the duration of IH cycles, ranging from 120 cycles/h (30 s cycle; Fletcher et al., 1992a; Julien et al., 2003; Dematteis et al., 2008), 80 cycles/h (6 min cycle; Knight et al., 2011), 60 cycles/h (1 min cycle; Campen et al., 2005), and when the chambers are larger, longer cycles are often used, reducing the number of cycles/h (Zoccal et al., 2007, 2008; Silva and Schreihofer, 2011) of daytime exposure, from 4 h/day (Kalaria et al., 2004), 6 h/day (Lai et al., 2006), 7 h/day (Fletcher et al., 1992a), 8 h/day (Chen et al., 2005; Belaidi et al., 2009; Zoccal et al., 2008, 2009; Knight et al., 2011; Silva and Schreihofer, 2011; Dyavanapalli et al., 2014; Schulz et al., 2014), 10 h/day (Liu et al., 2013) to 12 h/day (Lin et al., 2007). The exposure duration of 8 h/day seems to be that on which there is the greatest consensus (see Table 2). The duration of exposure seems to affect the study outcomes more than the hypoxic nadir or the rate of hypoxic cycling (Davis and O'Donnell, 2013).

An advantage of CIH models is they allow exposures that can be extended over months, enabling the investigation of chronic consequences that might occur in humans (Toth and Bhargava, 2013). The number of days necessary to induce an increase in BP seems to be dependent on the CIH paradigm. Some authors suggest that the BP increase triggered by CIH represents a time-dependent effect (Prabhakar et al., 2001; Hui et al., 2003; Dematteis et al., 2008; Zoccal et al., 2009). Moreover, both the time and severity of hypoxia have been shown to play an important role in the cardiovascular response (Li et al., 2007; Perry et al., 2007). It has recently been shown that a period of 14 days is not long enough to induce structural changes in cardiovascular structures, but these are already apparent after 35 days of incubation (Dematteis et al., 2008). Moreover, Iturriaga et al. report that the exposure of rats to CIH for 14 days enhanced the ventilatory response to hypoxia and produced a significant shift in heart rate variability, but these cardiorespiratory alterations occurred without noticeable changes in mean arterial BP until 21 days of CIH exposure (Iturriaga et al., 2010). Whereas some short-term protocols (7–14 days) cause a significant increase in BP (Belaidi et al., 2009; Knight et al., 2011; Silva and Schreihofer, 2011; Bathina et al., 2013), others show an increase in BP that occurs only after long-term exposure (35 days) to CIH (Prabhakar et al., 2001, 2005; Chen et al., 2005; Zoccal et al., 2009) (see Table 2). Finally, most IH paradigms in rodents do not include CO_2_ supplementation (Fletcher et al., 1999; Lin et al., 2007; Iturriaga et al., 2010; Perry et al., 2011; Bathina et al., 2013). In fact, only some authors have manipulated the CO_2_ levels (Ooi et al., 2000; Kantores et al., 2006; Dyavanapalli et al., 2014) and fixed the values along the protocol (see Table 2).

Independently of the paradigm used to induce HT related to OSA, previous reviews are unanimous in reporting the development of mild HT, despite the divergent changes in arterial blood gases (Kanagy, 2009) (see Table 2). The exceptions found in this review (Kalaria et al., 2004; Belaidi et al., 2009; Iturriaga et al., 2010; Perry et al., 2011) are all related to the method used for BP measurement. It is apparent that arterial catheterization is not an accurate method of measuring BP in CIH models. The methods most often used for BP measurement (for a review, see Kurtz et al., 2005) in IH models (see Table 2) are the tail-cuff method (Allahdadi et al., 2005; Chen et al., 2005; Soukhova-O'Hare et al., 2008; Belaidi et al., 2009; Totoson et al., 2013), radiotelemetry (Fletcher, 2000; Tahawi et al., 2001; Lai et al., 2006; Knight et al., 2011; Bathina et al., 2013; Sharpe et al., 2013; Dyavanapalli et al., 2014; Schulz et al., 2014), and arterial catheterization (Kanagy et al., 2001; Kalaria et al., 2004; Campen et al., 2005; Lin et al., 2007; Belaidi et al., 2009; Zoccal et al., 2009; Iturriaga et al., 2010; Perry et al., 2011; Silva and Schreihofer, 2011; Totoson et al., 2013).

### Humans

The variety of models of IH in healthy human subjects is much less impressive than that observed for animal models of sleep apnea. In terms of the exposure time, these models are usually divided into short-term and chronic (Foster et al., 2007). In short-term IH models, generally the exposure time (20–60 min) and the duration of the hypoxia or voluntary apnea period (30 s) are very limited. The protocols of Cutler et al. and Tamisier et al. are good examples of short-term models (Cutler et al., 2004; Tamisier et al., 2009). In contrast, Foster et al. made use of a chronic model, exposing healthy human volunteers to an hour of IH (5 min hypoxia alternating with 5 min normoxia) daily for 2 weeks (Foster et al., 2005). As in the animal models of IH, only some studies have controlled the level of CO_2_ (Foster et al., 2005), whereas others have not (Tamisier et al., 2009). Regardless of the protocol followed, exposing humans to CIH implies careful supervision.

In 2001, Xie et al. exposed nine healthy human subjects during wakefulness to 20 min of isocapnic hypoxia (arterial O_2_ saturation, 77–87%) and 20 min of normoxic hypercapnia (end-tidal PCO_2_, 15.3–8.6 Torr above eupnea) on two separate days. The subjects breathed through a leak-free nasal mask and the neurocirculatory and ventilatory responses to these two stimuli were further evaluated (Xie et al., 2001). These authors found that hypoxia induced a sympathetic activation that outlasted the chemical stimulus, whereas hypercapnia evoked a short-lived sympathetic activation (Xie et al., 2001). Years later, in a study performed with a larger sample (*n* = 31), Cutler et al. used a model of IH induced by voluntary apnea (30 s of hypoxic apnea every 1 min—simulating an AHI of 60/h—for 20 min) to determine if the cessation of breathing is important in prolonged sympathetic activation (Cutler et al., 2004). This study also included two other groups that were exposed to intermittent hypercapnic hypoxia and to intermittent isocapnic/hypoxia, respectively (Cutler et al., 2004). Their results support the hypothesis that short-term exposure to intermittent hypoxic apnea results in sustained elevation of post-ganglionic muscle sympathetic nerve activity and that hypoxia is the primary mediator of this response (Cutler et al., 2004). The data reported by Leuenberger et al. one year later were in line with these results (Leuenberger et al., 2005). They also found, in a study that enrolled 26 patients, a sustained sympathetic activation and also a transient elevation of BP following 30 min of voluntary end-expiratory apneas primed with a hypoxic gas mixture and lasting for 20 s in each minute (Leuenberger et al., 2005).

Foster et al. carried out three main studies in healthy human volunteers. The first aimed to determine the ventilatory, cardiovascular and cerebral tissue oxygen response to two protocols of IH (Foster et al., 2005). This study involved 18 patients randomly assigned to short-duration IH (1 h of 12% O_2_ separated by 5 min of normoxia) or long-duration IH (30 min of 12% O_2_). Both groups had 10 exposures over 12 days. Their findings show a rise in mean arterial blood pressure (MAP) that occurs throughout the daily exposure to short-duration IH but not during exposure to long-duration IH; moreover, they demonstrate that the vascular processes required to control blood flow and O_2_ supply to cerebral tissue in a healthy human are delayed following exposure to 12 days of isocapnic IH (Foster et al., 2005). In 2009, the same group reinforced the enrollment of IH on the pathogenesis of cardiovascular and cerebrovascular disease in patients with OSA (Foster et al., 2009). They exposed 10 healthy subjects to IH (2 min of hypoxia: nadir P_ET,O2_ = 45.0 mmHg, alternating with 2 min of normoxia: peak P_ET,O2_ = 88.0 mmHg for 6 h) for 4 consecutive days and concluded that IH alters BP (MAP increased by 4 mmHg) and induces an increase in cerebral vascular resistance (Foster et al., 2009). More recently, Foster et al. have assessed the role of the type I angiotensin II receptor in mediating an increase in arterial pressure associated with a single 6-h IH exposure (Foster et al., 2010). For that, they exposed nine healthy subjects to sham IH, IH with placebo medication, and IH with the type I angiotensin II receptor antagonist (losartan). Their findings demonstrate a significant increase in arterial pressure after exposure to isocapnic IH (Foster et al., 2010). Furthermore, since this increase is prevented by the blockade of AT_1_ receptors, these results suggest an important role for the rennin-angiotensin-aldosterone system (RAAS) in the pathophysiology of HT related to OSA (Foster et al., 2010).

Tamisier et al. have developed a novel model of nocturnal CIH in healthy humans, which represents an important step forward in the field, designed to overcome some of drawbacks and confounding factors that are present in studies of both animals and OSA patients (Tamisier et al., 2009). To investigate the effects of CIH on sleep, BP and ventilatory control, these authors make use of altitude tents to mimic the cyclical arterial oxygen desaturations-resaturations of sleep apnea. They delivered O_2_ for 15 s every 2 min during sleep while subjects breathed 13% O_2_ in a hypoxic tent to create 30 cycles/h of cyclic desaturation-reoxygenation (SpO_2_ range: 95–85%), and exposed subjects overnight for 8–9 h/day for 2 or 4 weeks (Tamisier et al., 2009). Among other results, they show that waking normoxic arterial pressure increased significantly at 2 weeks for systolic and for diastolic at 4 weeks, that patients developed a sustained BP increase during the day and exhibited a steeper BP decrease at night compared to baseline BP values, and finally, that this model produces clinically relevant fluctuations in SaO_2_ (Tamisier et al., 2009). Although undoubtedly relevant, the authors recognize the presence of several respects in which their model does not mimic sleep apnea, e.g., no negative intrathoracic pressure development, higher percentage of sleep time at <90% SaO_2_ and poikilocapnia (Tamisier et al., 2009). However, some of these limitations can be overcome to achieve a pattern of IH more akin to OSA features. This model was further used by the same group in 2011 to shed light on the profile of the BP increase previously described to determine if it is sustained and to explore potential underlying physiological mechanisms. The authors found that only 2 weeks of severe IH exposure produces a sustained daytime BP increase in the setting of sympathetic activation and blunted vascular sympathic baroreflex gain in healthy volunteers (Tamisier et al., 2011).

In conclusion, to date, only a small number of studies have been conducted using healthy human models of IH and these have primarily been aimed at elucidating the role of IH in sustained sympathetic activation and cerebrovascular regulation. Only a few studies have evaluated BP outcomes (Foster et al., 2009, 2010; Tamisier et al., 2009, 2011) and none of these models have truly been used to assess the efficacy of AHDs in the treatment of HT related to OSA. In fact, in the later work of Foster et al., losartan (the angiotensin II AT_1_ receptor antagonist) was used only to demonstrate a mechanistic pathway rather than to evaluate its efficacy (Foster et al., 2010). Thus, future research in this field is clearly needed.

## What are the mechanisms involved in the pathogenesis of HT related to OSA?

Fletcher et al. were pioneers in demonstrating the hypertensive effect of CIH (Fletcher et al., 1992a) and the role of the sympathetic nervous system, peripheral receptors and rennin-angiotensin system in this response (Fletcher et al., 1992b, 1999, 2002; Fletcher, 2000). This group also showed that surgical denervation of peripheral chemoreceptors, adrenal demedullation and chemical denervation of the peripheral nervous system prevented the increase in BP in response to CIH stimulus (Fletcher et al., 1992b; Bao et al., 1997). After Fletcher et al.'s first work, many reports enabled confirmation of the relationship between IH and BP increases and contributed to elucidating the underlying mechanisms. Kanagy et al. reported increased plasma endothelin-1 levels in rats exposed for 11 days to CIH, which also demonstrated an appreciable increase in MAP (Kanagy et al., 2001). In 2006, Lai et al. suggested that chronic IH-induced sustained HT was associated with the facilitation of cardiovascular sympathetic outflow followed by decreases in baroreflex sensitivity in conscious rats (Lai et al., 2006). Along the same line, the work undertaken by Zoccal et al. provided strong evidence to support the idea that rats submitted to CIH show an increase in sympathetic activity, which seems to be essential in the maintenance of high BP values in the CIH model (Zoccal et al., 2007). Another group revealed that although elevated sympathetic nervous system activity (SNA) may contribute to CIH-induced HT, reduced adrenergic vascular reactivity buffers the cardiovascular impact of exaggerated acute raises in SNA (Silva and Schreihofer, 2011). Data attained by Knight et al. indicated that CIH induces an increase in FosB/ΔFosB in autonomic nuclei and suggested that activator protein-1 (AP-1) transcriptional regulation may contribute to stable adaptative changes that support chronically elevated BP (Knight et al., 2011). Also in 2011, Liu et al. demonstrated that CIH activates the HIF-1α/endothelin system, through CIH-NADPH oxidase-mediated ROS production, and this enhances the development of resistant vasoconstriction and elevates BP in rats (Liu et al., 2011). The study undertaken by Bathina et al. revealed that the knockdown of tyrosine hydroxylase in the nucleus of the solitary (NTS) tract reduces the CIH-induced persistent increase in MAP, suggesting that noradrenergic A_2_ neurons in nucleus tractus solitarius play a role in the cardiovascular responses to CIH (Bathina et al., 2013). More recently, Schulz et al. have shown that NADPH oxidase 2 (NOX2) knockout blocks the development of HT induced by CIH, suggesting that this type of HT is mediated by reactive oxygen species (ROS) derived from the activation of NOX2 within cells located outside the cardiovascular system (Schulz et al., 2014).

The mechanisms involved in the genesis of HT related to OSA have recently been reviewed (Lavie and Lavie, 2009; Bosc et al., 2010; Sunderram and Androulakis, 2012; Zhang and Si, 2012; Lévy et al., 2013) and broadly include the following: sympathetic nervous system stimulation mediated mainly by the activation of carotid body chemoreflexes, decreased vascular responses to nitric oxide, increased plasma concentrations of endothelin, and elevation of proinflammatory cytokines (TNF-α, IL-6, VEGF). While for some of these mechanisms (e.g., activation of the RAAS, endothelial dysfunction, systemic inflammation, metabolic anomalies, and genetic contribution) the relationship with OSA and subsequent cardiovascular morbidity remain partially unclear and there is a need to gather more evidence, for others (e.g., the increase in sympathetic activity and acute effects of negative intrathoracic pressure), there seems to be more agreement on the linkage and it is well-documented (Parati et al., 2013). In fact, based on data attained from patients with OSA, it is widely accepted that sympathetic activation, inflammation and oxidative stress play major roles in the pathophysiology of this particular type of HT. In addition, the use of animal models has revealed that CIH is the critical stimulus underlying sympathetic activity and HT, and that this effect requires the presence of functional arterial chemoreceptors (Fletcher, 2000). However, it should be also mentioned that HT related to OSA probably results not only from increased carotid chemoreflex but also from decreased baroreceptor activity (Dumitrascu et al., 2013). It is also important to highlight the potential role of obesity as an intermediate factor in the pathway of HT related to OSA (Young et al., 2007; O'Connor et al., 2009).

**Figure 1:**
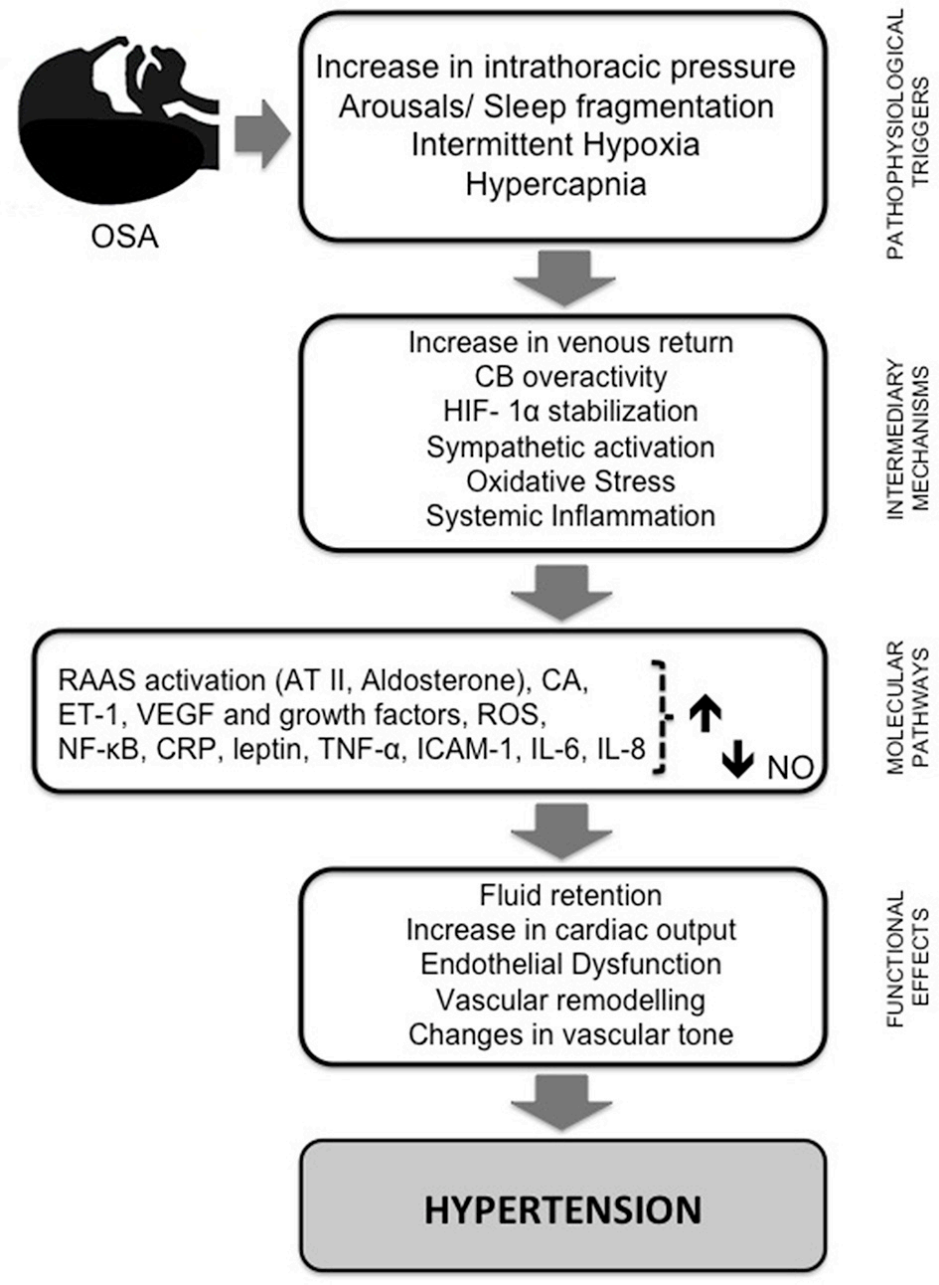
**Schematic diagram summarizing the pathways by which intermittent hypoxia leads to hypertension**. Repetitive obstructive apneas or hypopneas lead to increased intrathoracic pressure, sleep fragmentation, recurrent hypercapnia, and intermittent hypoxia (IH). This last phenomenon plays a pivotal role in triggering several intermediary mechanisms and molecular pathways that contribute to the initiation and progression of cardiac and vascular pathology. First, IH enhances sympathetic nervous system activity, leading to vasoconstriction and systemic hypertension through RAAS activation, and an increase in catecholamine secretion and plasma level of vasoconstrictive ET-1. Episodic hypoxia also favors the stabilization of HIF-1α and the production of ROS, which is followed by increased expression of NF-κ B and decreased NO bioavailability, the most important vasodilatory molecule synthesized by the endothelium. AT II and ET-1 both seem to be implicated in vascular remodeling and ROS formation, which is increased through the activation of vascular NADPH oxidase and xanthine oxidase. ROS molecules induce a cascade of inflammatory pathways linked to an overexpression of adhesion molecules and pro-inflammatory cytokines, and oxidative stress may trigger sympathetic hyperactivation and *vice versa*. ROS production is required for HIF-1α induction and HIF-1α induction is required for ROS production. In addition, HIF-1α promotes the expression of ET-1 and transcriptional activation of VEGF and other growth factors. Activation of NF-κ B also seems to be central in inflammation induced by IH due to its regulatory role in the production of pro-inflammatory mediators (e.g., TNF-α, IL-6, IL-8, ICAM-1, and CRP). These signaling pathway proteins, combined with RAAS, decreased expression of eNOS, and increased ROS production and stabilization of HIF-1, participate in the molecular mechanisms underlying the endothelial dysfunction induced by IH. Together, these mechanisms progress to fluid retention, changes in cardiac output and vascular tone, and vascular remodeling, leading to systemic HT, one of the major consequences of OSA. AT II, angiotensin II; CA, catecholamine levels; CRP, C- reactive protein; CB, carotid body; ET-1, endothelin 1; HIF-1α, hypoxia-inducible factor α; IH, intermittent hypoxia; IL, interleukin; ICAM-1, intercellular adhesion molecule; NO, nitric oxide; eNOS, endothelial nitric oxide synthase; NF-κ B, nuclear factor-κ-light chain enhancer of activated B cells; RAAS, renin-angiotensin-aldosterone system; ROS, reactive oxygen species; TNF- α, tumor necrosis factor α; VEGF, vascular endothelial growth factor.

The mechanisms involved in the pathogenesis of HT can be summarized in relation to two main pathways: sympathetic nervous system stimulation mediated mainly by activation of carotid body (CB) chemoreflexes and the systemic effects of CIH, mainly due to the activation of NOX2 and subsequent ROS production. Figure 1 illustrates the hypothesized pathways by which intermittent hypoxia leads to HT.

## What is already known concerning the efficacy of AHDs?

### Humans

Despite the considerable number of studies involving OSA patients, only a few have investigated the efficacy of different AHDs and in general, they tend to be individual drug studies. Moreover, most of the studies only take into account the number of drugs taken by patients to adjust this variable and are difficult to interpret as most of the patients were already under AHDs regimens. This lack of information could be attributed to the large number of possible different AHDs regimens observed in OSA patients. Table 3 summarizes the most relevant studies that have investigated the efficacy of AHDs in OSA patients.

**Table 3:** **Studies of the efficacy of AHDs in OSA patients**.

**Study design**	**n**	**CPAP (Y/N)**	**AHDs; dosage (mg/day)**	**BP measurement**	**BP outcome**	**References**
RCT; double-blinded; balanced incomplete block design (6 w each drug + 3 w washout)	40	No	Atenolol (50); amlodipine (5); enalapril (20); hydrochlorothiazide (25); losartan (50)	Office BP 24 h ABPM	↓ in office SBP and daytime ABPM NS for all drugs; Atenolol ↓ night-time 24 h SBP and DBP more effectively than amlodipine, enalapril or losartan	Kraiczi et al., 2000
RCT; double-blinded; crossover schedule (8 w each drug + 2–3 w washout	15	No	Atenolol (50); isradipine (2.5): hydrochlorothiazide (25); spirapril (6)	Office BP	Slight ↓ BP for all drugs; Only atenolol affected BP variability	Salo et al., 1999
RCT; double-blinded; crossover (8 w each drug + 2–3 w washout	18	NA	Atenolol (50); isradipine (2.5); hydrochlorothiazide (25); spirapril (6)	24 h ABPM	↓ mean 24 h SBP (except for HCTZ) ↓ mean 24 h DBP (for all drugs) NS ↓ mean night-time SBP and DBP (for all drugs)	Pelttari et al., 1998
RCT (3 months each treatment)	75	Yes	Treatment with at least 3 drugs at adequate doses, including a diuretic	24 h ABPM	CPAP + AHDs regimen: ↓ 4.9 mmHg 24 h DBP; AHDs regimen alone: NS	Lozano et al., 2010
RCT; single-blinded (3 w each regimen)	44	Yes	Valsartan (160) + amlodipine (5–10) + hydrochlorothiazide (25)	Office BP 24 h ABPM	AHDs alone: ↓ office and 24 h SBP and DBP Additional ↓ in office BP and ambulatory BP monitoring (CPAP+ 3 AHDs)	Litvin et al., 2013
RCT; crossover (8 w each treatment + 4 w washout)	23	Yes	Valsartan (160)	Office BP 24 h ABPM	CPAP: ↓ 2.1 mmHg 24 h MBP and ↓ 1.3 mmHg night-time MBP (NS) VAL: ↓ 9.1 mmHg 24 h MBP and ↓ 6.1 mmHg night-time MBP	Pépin et al., 2009
RCT (8 w)	12	No	Spironolactone (25–50) added to current medication (mean number of AHDs: 4.3 (*SD* = 1.1)	Office BP 24 h ABPM	↓ 17 mmHg 24 h SBP ↓ 10 mmHg 24 h DBP	Gaddam et al., 2010
RCT; double-blinded (8 days)	12	NA	Metoprolol (100); cilazapril (2.5)	Office BP 24 h ABPM	MET: ↓ 13 mmHg 24 h SBP and ↓ 5 mmHg 24 h DBP CIL: ↓ 13 mmHg 24 h SBP and ↓ 17 mmHg 24 h DBP	Mayer et al., 1990
RCT; double-blinded; crossover (2 w each treatment + 3 w washout)	16	No	Doxazosin (4–8); enalapril (10–20)	24 h ABPM	DOX: ↓ 4.1 mmHg 24 h SBP and ↓ 5.1 mmHg 24 h DBP EN: ↓ 12.6 mmHg 24 h SBP and ↓ 8.9 mmHg 24 h DBP 24 h MBP: no differences between groups	Zou et al., 2010
RCT; double-blinded; parallel group; single center (6 w)	31	No	Nebivolol (5); valsartan (80)	Office BP	NEB: ↓ 14.6 mmHg SBP and ↓ 8.6 mmHg DBP VAL: ↓ 11.6 mmHg SBP and ↓ 8.9 mmHg DBP No differences between treatments	Heitmann et al., 2010
RCT; prospective; crossover; parallel group (2 single doses of each drug + 2 w washout)	11	No	Nifedipine slow-release (40); carvedilol (20)	Office BP TSP method	NIF: ↓ 24.2 mmHg mean SBP and ↓ 18.7 mmHg mean DBP CAR: ↓ 16 mmHg mean SBP and ↓ mean 8.6 mmHg DBP	Kario et al., 2014
RCT; double-blinded; placebo-controlled (8 days)	23	NA	Cilazapril (2.5)	Invasive arterial BP (arteria brachialis)	↓ 10 mmHg MBP (vs. ↓ 4.3 mmHg MBP for placebo)	Grote et al., 1994

*ABPM, ambulatory blood pressure monitoring; AHDs, antihypertensive drugs; BP, blood pressure; CAR, carvedilol; CIL, cilazapril; CPAP, continuous positive airway pressure; DBP, diastolic blood pressure; DOX, doxazosin; EN, enalapril; HCTZ, hydrochlorothiazide; MBP, mean blood pressure; MET, metoprolol; NA, information not available; NEB, nebivolol; NIF, nifedipine; NS, no significant effect; RCT, randomized controlled trials; SBP, systolic blood pressure; SD, standard deviation; TSP, trigger sleep BP monitoring; VAL, valsartan; w, week; ↓, decrease*.

In a study undertaken by Pelttari et al., the AH effects of four different AHDs (atenolol: a beta-blocker; isradipine: a calcium channel blocker; hydrochlorothiazide: a diuretic; spirapril: an angiotension-converting enzyme inhibitor) in obese patients with OSA and HT were compared using ambulatory blood pressure monitoring (ABPM) (Pelttari et al., 1998). This study revealed that although daytime HT was quite easily controlled by the single use of these drugs (especially with atenolol and isradipine; diuretics did not significantly lower BP) none of the AHDs were able to produce a significant decrease in nocturnal BP (Pelttari et al., 1998). Mayer et al. carried out another comparative study between cilazapril (an angiotension-converting enzyme inhibitor) and metoprolol (a beta-blocker) (Mayer et al., 1990). Their findings showed that despite the short period of therapy (1 week), both metoprolol and cilazapril lowered nighttime BP in OSA patients (Mayer et al., 1990).

A multiple crossover study examined the BP-lowering effect of the five major AHDs classes (atenolol: beta-blocker; amlodipine: calcium channel blocker; enalapril: angiotension-converting enzyme inhibitor; hydrochlorothiazide: diuretic; losartan: angiotensin receptor blocker) and showed that atenolol induced the most pronounced effect in lowering BP (Kraiczi et al., 2000). Atenolol was more efficient in reducing mean nighttime diastolic and systolic BP (measured by ABPM) compared to amlodipine, enalapril, hydrochlorothiazide, and losartan (Kraiczi et al., 2000). Salo et al. investigated the effects of four AHDs (atenolol; isradipine: a calcium channel blocker; hydrochlorothiazide; spirapril: an angiotension-converting enzyme inhibitor) on cardiovascular autonomic control and reactivity in HT OSA patients (Salo et al., 1999). This group reported that of the four drugs, only atenolol effected BP variability (Salo et al., 1999). Thus, the results of these two pilot studies are in line with those arguing the involvement of the sympathetic system in the pathophysiology of HT related to OSA, suggesting that beta-blockers, in particular atenolol, may have beneficial effects beyond BP reduction in patients with OSA. However, both studies presented low levels of causation, which could have limited the ability to detect differences between classes.

Nevertheless, it has been advanced that angiotension-converting enzyme inhibitors (ACEi) treatment could exacerbate OSA by inducing upper airway inflammation (Cicolin et al., 2006). The comparison between chronic treatments of ACEi and angiotensin AT1 receptor antagonists in terms of AH efficacy and levels of inflammatory markers has never been performed either in humans with OSA or in animal models. More recently, other study compared the effect of doxazosin (an α1- adrenergic receptor antagonist) and enalapril (an angiotensin-converting enzyme inhibitor) on nocturnal BP control and concluded that the former has a proportionally poorer effect than the latter (Zou et al., 2010). In 1994, Grote et al. performed a study aimed at assessing the effectiveness of cilazapril (an angiotension-converting enzyme inhibitor) in managing high BP in patients with OSA. Although the study comprised a small sample size, the results suggested that cilazapril is effective in reducing BP in all sleep stages (Grote et al., 1994). In another small study, Heitmann et al. evaluated the effect of nebivolol (a third generation beta-blocker) on BP reduction and sleep apnea activity in HT patients with mild to moderate OSA in comparison with valsartan (an angiotensin receptor blocker) and concluded that the effect of these AHDs were similar (Heitmann et al., 2010). Despite the same limitations, these studies highlight the role of the renin-angiotensin-aldosterone system (RAAS) in the pathophysiology of HT related to OSA.

In two past studies (Lozano et al., 2010; Litvin et al., 2013), patients either received CPAP in combination with AHDs or alternatively, the pharmacological treatment alone, allowing the evaluation of the effects of CPAP and AHDs independently or in conjunction. In the study undertaken by Lozano et al., patients were under an AHDs regimen with at least three drugs at adequate doses, including a diuretic (Lozano et al., 2010). The authors noted a significant decrease in the mean 24-h diastolic BP in patients who received CPAP in addition to conventional treatment, suggesting that resistant HT treated with both CPAP and AHDs provides greater BP reduction than AHDs alone (Lozano et al., 2010). However, in patients who used CPAP less than the average (5.6 ± 1.52 h/night) and for those treated with conventional treatment alone, there was no significant difference in the 24-h ambulatory BP values (Lozano et al., 2010). These findings are in line with those reported by Litvin et al., attained with patients who received stepped dose titration of AHDs treatment (valsartan 160 mg + amlodipine 5–10 mg + hydrochlorothiazide 25 mg) for 3 months before CPAP was added (Litvin et al., 2013). These findings seem to suggest that the best strategy to treat HT related to OSA involves the combination of OSA treatment with CPAP and the use of AHDs. This combination is likely to be more effective in lowering both daytime and nighttime BP than either treatment alone (Phillips and O'Driscoll, 2013). In addition, Pépin et al. explored RAAS inhibition using losartan in a crossover randomized control trial. In this study, the authors compared the efficacy of CPAP and valsartan in reducing BP in HT patients with OSA never treated for either condition (Pépin et al., 2009). They reported that although the BP decrease was significant with CPAP treatment, valsartan induced a four-fold higher decrease in mean 24-h BP than CPAP in this specific sample (Pépin et al., 2009).

In an earlier report, 74 of the 393 OSA patients using AH medications on a regular basis for more than 6 months were deemed to have been treated “ineffectively” (Lavie and Hoffstein, 2001), but the characterization of these medications was not reported. The same limitation is found in the study of Deleanu et al., which aimed to study the effect of medication-controlled HT on OSA patients (Deleanu et al., 2014). The authors suggested that controlled BP abates sleepiness and reduces remaining symptoms (e.g., headaches, impotence and morning fatigue). These findings could be much more interesting if the regimens responsible for these effects were revealed.

In a very recent study, Kario et al. aimed to evaluate the effects of bedtime dosing of vasodilating (nifedipine, a calcium channel blocker) vs. sympatholytic (carvedilol, a non-selective β-blocker/α_1_-blocker) AH agents on the sleep BP profile in HT OSA patients (Kario et al., 2014). For this, they made use of a new BP monitoring method, the trigger sleep BP monitoring (TSP) method, which is based on the automated fixed-interval measurement function with an additional oxygen-triggered function that initiates BP measurement when oxygen desaturation falls below a set variable threshold continuously monitored by pulseoximetry (Kario et al., 2014). The BP lowering effects of nifedipine on the mean and minimum sleep systolic BP were stronger than those of carvedilol; moreover, sleep systolic BP surge (the difference between the hypoxia peak systolic BP—SBP—measured by the oxygen-triggered function and SBP within 30 min before and after the peak SBP) was only significantly reduced by carvedilol (Kario et al., 2014). Thus, both drugs are effective in decreasing sleep BP (Kario et al., 2014) but the effect of carvedilol seems to be related more specifically to the hypoxia stimuli than nifedipine.

Finally, Cichelero et al. recently published the protocol of their randomized double-blind clinical trial, which seeks to compare the efficacy of chlorthalidone (a diuretic) with amiloride (also a diuretic) vs. amlodipine (a calcium channel blocker) as a first drug option in patients older than 40 years of age with stage I HT and moderate OSA (Cichelero et al., 2014). The findings of this study have not yet been reported.

In summary, individual drug studies find that the blockade of β1-adrenergic receptors (e.g., atenolol and nebivolol) and the renin-angiotensin-aldosterone (RAA) pathway, including both ACEi and angiotensin AT1 receptor antagonists, might be helpful. Spironolactone (a mineralocorticoid receptor antagonist) has been proposed has a very useful tool in cases of resistant HT (Ziegler et al., 2011a,b), a very prevalent condition in OSA patients (Oliveras and Schmieder, 2013; Solini and Ruilope, 2013) in which aldosterone levels are generally elevated, as well as for severe OSA patients (Ziegler et al., 2011a). Moreover, a study performed by Gaddam et al. (2010) has provided preliminary evidence that treatment with this drug substantially reduces the severity of OSA and improves BP in patients with both OSA and resistant HT (Gaddam et al., 2010). These results seem promising but need to be confirmed in further larger studies. In contrast, despite volume overload appears to play a large role in the development of OSA (Owen and Reisin, 2013), diuretics, namely thiazide, have not been very effective AH agents in OSA patients without fluid retention (Ziegler et al., 2011b). Calcium channel blockers, although effective in lowering BP, seem to present an effect less related to hypoxia stimuli. Moreover, Nerbass et al. reported that the use of these drugs might impact negatively on sleep duration in HT patients with OSA (Nerbass et al., 2011). They reported that the use of calcium channel blockers was associated with significant reduction in total sleep time and lower sleep efficiency (Nerbass et al., 2011). Thus, their prescription can be questionable in these patients.

Despite the findings of these studies, they present some limitations and important data are missing. The major limitations comprise the following: the variability of subjects included in the studies as most of them were performed in non-AHDs naïve patients; the severity and chronicity of HT, which were not taken into account and consenquently the clinical relevance of BP reduction is questionable; the drug effectiveness in reducing nocturnal BP, which was not assessed in some studies; the confounding risk factors for HT that might be present in OSA patients (e.g., obesity) and were not properly addressed in most studies. Furthermore, we can point out several questions that are still unanswered, e.g., how many OSA patients are controlled under monotherapy with beta- blockers, angiotensin-converting enzyme inhibitors (ACEis), and angiotensin II receptor blockers (ARBs)? Beta-blockers or RAAS blockers are apparently effective, but should they be used alone or in combination? How many OSA patients remain uncontrolled despite the use of two or more AHDs? How do different AHDs behave when included in an AHDs regimen? In addition, the impact of these studies in clinical practice is unknown because epidemiological studies designed to investigate the AH medication profile in OSA patients are lacking. In addition, the more recent recommendations for the management of patients with OSA and HT are inconclusive regarding the use of AHDs and recognize the lack of strong evidence for the establishment of a first-line AHDs regimen for these patients (Parati et al., 2013). Other authors support the idea that as there is no clear evidence for prefering a specific class of AHDs, the selection should primarily be guided by the patient's cardiometabolic profile and associated comorbidities (e.g., obesity, metabolic syndrome, diabetes mellitus, and cardiovascular diseases) (Tsioufis et al., 2010). Moreover, these authors recommend that due to the lack of relevant trials focused on the use of associations of AHDs in OSA patients, the choice should rely on current HT guidelines and the adverse effects of AHDs also need to be considered (Tsioufis et al., 2010). The limited evidence base restricts the ability to make informed treatment choices. Thus, larger scale observational and clinical studies are needed to address these and possibly other limitations and bring new insights to the field.

Another problem concerning the studies carried out in humans is that HT is frequently not recognized in patients with OSA (Baguet et al., 2009), and it is important to highlight that patients with elevated BP who do not carry the diagnosis of HT may be misclassified as non-HT (Wang and Vasan, 2005). Consequently, aggressive control of BP must be warranted in OSA patients and an accurate method for BP measurement should be used in the early diagnosis of clinically suspected OSA patients. Taking into account the advantages and limitations of the several methods of BP measurement, 24-h ABPM seems to be superior to office BP measurement and home BP monitoring in diagnosing HT in patients with suspected OSA (for a review, see Parati et al., 2012).

### Animals

As previously stated, a rather wide variety of animal models has been used to evaluate the cardiovascular consequences of OSA and to study the cause-effect mechanisms in OSA. As CIH causes a moderate increase in BP, drugs can be tested further to modulate this effect. However, studies aimed at investigating the AH effect of drugs on animal models are scarce. Table 4 summarizes the studies that have evaluated the effects of AHDs on BP in animal models of CIH.

**Table 4:** **Studies evaluating the effects of AHDs on BP in animal models of CIH**.

**Species**	**CIH experimental protocol**	**Drugs/ intervention**	**BP measurement**	**Drug effect on BP**	**References**
Sprague-Dawley rats	2/3–20.9% O_2_ (3–6 s + 15–18 s; 2 cycles/min); 6–8 h/day; 35 days	Losartan (15 mg/kg); gavage; 35 days	Telemetry	Significant ↓ MAP (98.2 ± 61.7 to 85.9 ± 62.7 mm Hg)	Fletcher et al., 1999
Sprague-Dawley rats	5–21% O_2_+ 5–0%CO_2_ (20 cycles/h); 7 h/day; 14 days	A-779 (Ang-(1–7) antagonist); Losartan (2 nmol/h) and ZD7155 (AT1 antagonists); PD123319 (AT2 receptor antagonist); osmotic minipumps delivered into PVN; 14 days.	Telemetry	↓ MAP: A-779: 5 ± 1 mm Hg, Losartan: 9 ± 4 mmHg, ZD7155: 11 ± 4 mmHg PD123319: 4 ± 3 mmHg	da Silva et al., 2011
Sprague-Dawley rats	20.9–10% (180 s cycles); 10 h/day; 35 days	Losartan (15 mg/kg); p.o. (syringe technique); 35 days	*NA* (arterial catheterization?)	↓ SBP (10 mmHg)	Fenik et al., 2012
Sprague-Dawley rats	80 cycles (6 min each) 21–10% O_2_/day; 8 h/day; 7 days	Losartan (1 μg/h); intracerebroventricular (miniosmotic pumps); 7 days	Telemetry	↓ MAP during both CIH exposure and normoxic period	Knight et al., 2013
Sprague-Dawley rats	20 cycles (90 s each) of 21–5% O_2_ and 0–5% CO_2_/h; 7 h/day; 14 days	BQ-123 (10–1000 nmol/kg in bolus or 100 nmol/kg/day for chronic administration); iv or sc; 14 days	Tail-cuff method and telemetry	Acute administration: dose dependent ↓ MAP Chronic administration: prevented ↑MAP	Allahdadi et al., 2008
SHR + Wistar rats	21–10% O_2_ (1 min cycles: 20 + 40 s); 8 h/day; 14 days	Bosentan (100 mg/Kg/dia); mixed in chow; 14 days	Tail-cuff method + Arterial catheterization	Prevented ↑MAP	Belaidi et al., 2009
SHR	21–10% O_2_ (every 90 s); 12 h/day; 30 days	Nifedipine (5 mg/Kg) and SOD mimetic (MnTMPyP; 10 mg/Kg); s.c.; 30 days	Tail-cuff method	Nifedipine: attenuate SBP and DBP SOD mimetic: ↓ SBP and DBP	Soukhova-O'Hare et al., 2008
Sprague-Dawley rats	21–5% O_2_ (every 60 s); 8 h/day; 14–21 days	Melatonin (10 mg/Kg); i.p.; 14 or 21 days (30 min before hypoxic exposure)	Tail-cuff method	↓ SBP (21 mmHg)	Hung et al., 2013
Sprague-Dawley rats	20 cycles (90 s each) of 21–5% O_2_ and 0–5% CO_2_/h; 7 h/day; 14 days;	Tempol (1 mM); drinking water; 14 days	Telemetry	↓ MAP (17 mmHg)	Troncoso Brindeiro et al., 2007
Sprague-Dawley rats	12 cycles (300 s each) of 21–5% O_2_/h; 8 h/day; 21 days	Ascorbic acid (1.25 g/L); drinking tap water; 21 days	Arterial catheterization	↓ MAP (29 mmHg)	Del Rio et al., 2010
Sprague-Dawley rats	9 cycles (5 min + 15 s) of 21–5% O_2_/h; 8 h/day; 10 days	SOD mimetic (MnTMPyP; 5 mg/Kg/day); i.p; 10 days	Arterial catheterization	↓↓ MAP	Kumar et al., 2006
Sprague-Dawley rats	20 cycles (90 s each) of 21–5% O_2_ and 0–5% CO_2_/h; 8 h/day; 11 days	PD145065 (ET receptor antagonist in cumulative drugs: 0.3, 3.0, 30, 300, 1000 nmol/Kg); bolus; 11 days	Arterial catheterization	Dose dependent ↓ MAP	Kanagy et al., 2001
Sprague-Dawley rats	21–5% O_2_ (12 times/h); 8 h/day; 14 days	Ebselen (specific ONOO- scavenger; 10 mg/kg/day); osmotic mini-pumps; 7 days	Telemetry	↓ elevated BP	Moya et al., 2014

*AHDs, antihypertensive drugs; BP, blood pressure; CIH, chronic intermittent hypoxia; DBP, diastolic blood pressure; ET, endothelin; h, hour; HT, hypertension; i.p., intraperitoneal; MAP, mean arterial pressure; NA, information not available; min, minutes; p.o., per os; PVN, hypothalamic paraventricular nucleus; s, seconds; SBP, systolic blood pressure; SHR, spontaneously hypertensive rats; s.c., subcutaneous; SOD, superoxide dismutase mimetic; ↓, decrease; ↑, increase*.

In a study undertaken to clarify the role of renal sympathetic nerve activity and plasma renin activity (PRA) in the diurnal BP response to chronic IH, Fletcher et al. demonstrated that the pharmacological blockade of the RAAS with losartan prevented the rise in BP induced by CIH (Fletcher et al., 1999, 2002). Losartan and other angiotensin antagonists (A-779, an Ang-(1–7) antagonist; ZD7155, an AT1 antagonist; PD123319, an AT2 receptor antagonist) were further used by da Silva et al. (2011) to investigate the role of endogenous angiotensin peptides within the hypothalamic paraventricular nucleus (PVN) neurons to control BP in a rat model of CIH-induced HT. These authors concluded that endogenous angiotensin peptides acting in the PVN contribute to IH-induced increases in MAP observed in this rat model. In 2013, losartan was used once again to test the role of the brain RAAS in CIH HT (Knight et al., 2013). The work of this group provided evidence that brain RAAS contributes to CIH HT and that brain RAAS appears to be critical for the development and maintenance of the sustained HT during normoxia (Knight et al., 2013).

Other groups have found that the systemic administration of endothelin (ET) receptor antagonists in rodents prevents the increase in BP during CIH exposure (Kanagy et al., 2001; Allahdadi et al., 2008; Belaidi et al., 2009). The data provided by Allahdadi et al. showed that an endothelin receptor antagonist (ET_A_: BQ-123) acutely decreased the MAP dose dependently in rats exposed to IH but not sham rats, suggesting that targeting ET_A_ receptors may be a selective and effective treatment of HT related to OSA (Allahdadi et al., 2008). Belaidi et al. used SH rats and bosentan, a mixed endothelin receptor antagonist (Belaidi et al., 2009). Their results showed that the administration of bosentan during chronic IH prevented the increase in BP and reinforced the idea that endothelin antagonists could be useful therapeutic tools in HT related to OSA (Belaidi et al., 2009). The same effects were reported by Kanagy et al. for PD145065, a non-selective endothelin receptor antagonist (Kanagy et al., 2001).

Soukhova-O'Hare et al., designed a study based on the assumption that ROS and altered L-Ca^2+^ channel activity may underlie the post-natal programing of exaggerated BP and cardiac remodeling (Soukhova-O'Hare et al., 2008). To test this hypothesis, these authors used nifedipine, an L-calcium channel blocker, and a superoxide dismutase mimetic (MnTMPyP pentachloride); both attenuated BP (Soukhova-O'Hare et al., 2008). Their results suggested that Ca^2+^ and reactive oxygen species-mediated signaling during IH are critical mechanisms underlying post-natal programing of an increased severity of HT in SH rats. Kumar et al. reported similar results for the same superoxide dismutase mimetic (Kumar et al., 2006). A year before, Troncoso Brindeiro et al. used another superoxide dismutase mimetic, tempol, and showed that scavenging superoxide prevents both the increase in ET-1 production and vascular ROS levels induced by CIH exposure (Troncoso Brindeiro et al., 2007). The later work of Del Rio et al., using the antioxidant ascorbic acid, showed that this substance prevented the increased plasma peroxidation and nitrotyrosine formation within the carotid body, as well as HT (Del Rio et al., 2010), supporting the essential role of oxidative stress in the generation of carotid body chemosensory potentiation and systemic cardiorespiratory alterations induced by IH (Del Rio et al., 2010).

More recently, Hung et al. tested the hypothesis that melatonin, previously shown to ameliorate oxidative injury and inflammation, could have a protective effect against IH-induced HT and endothelial dysfunction. This assumption was confirmed as melatonin promoted a decrease in systolic BP and prevented endothelial dysfunction with ameliorated levels of nitric oxide, endothelial-dependent relaxation, and expressions of eNOS and antioxidant enzymes (Hung et al., 2013).

Based on the studies described, we can conclude that most reports on CIH animal models in which drugs have been tested were not designed to respond to pharmacological issues: they have been used solely as pharmacological tools to address physiological mechanisms. The experiments evaluate prevention but not the effectiveness of treatment. They must be planned first to induce HT and then evaluate the efficacy of cumulative doses of drugs because the translation of the results to humans obtained with simultaneous induction of HT and drug administration is not relevant. Other limitations of the pharmacological approaches included in these works are the absence of dose-response curves and comparison of the effectiviness of different drugs in the same animal model. Thus, other studies must be designed to overcome these drawbacks.

## What are the potential non-pharmacological approaches to the management of HT related to OSA?

Taken in perspective, the pathophysiology of HT related to OSA, which involves an increase in sympathetic activity, renal denervation seems to be a logical approach for patients with this type of HT as renal sympathetic nerves are involved in the regulation of BP. Indeed, the beneficial role of this novel approach in the management of resistant HT and other cardiovascular diseases has been reported and reviewed extensively (Grassi et al., 2012; Pimenta and Oparil, 2012; Böhm et al., 2013; Ukena et al., 2013; Urban et al., 2013; Faselis et al., 2014; Tsioufis et al., 2014). Moreover, Shantha and Pancholy (2014) have recently undertaken a systematic review of the effect of renal sympathetic denervation on AHI in patients with OSA. Curiously, they concluded that this approach is associated with a significant reduction in mean AHI (Shantha and Pancholy, 2014). However, as the authors pointed out, these results need further validation due to the low causal basis of the studies included in the analysis and due to the fact that only one of these studies was performed fully in a specific population of OSA patients; in the remaining studies, the diagnosis of OSA was only established after inclusion (Shantha and Pancholy, 2014). In a recent pilot study, the effect of renal denervation on BP control in patients with OSA was explored (Witkowski et al., 2011). Despite the low causal basis (*n* = 10), their findings demonstrated a significant BP decrease within 3 months, which was further enhanced at 6 months, exhibiting a drop pattern similar to clinical studies in resistant HT (Witkowski et al., 2011). Nontheless, further studies are needed to support this impressive effect of renal denervation and to ensure the safety of this technique for patients with HT related to OSA.

Like renal denervation, carotid baroreceptor stimulation has also been proposed as a novel AH therapy based on the recent evidence that baroreceptors might play an important role even in long-term BP regulation (Papademetriou et al., 2011; Grassi et al., 2012; Lovic et al., 2014). The main similarities and differences between these two novel approaches have been reviewed extensively by a group of Italian researchers (Grassi et al., 2014; Seravalle et al., 2014). Although electrical baroreflex stimulation appears to be safe and effective, and might represent a useful tool for managing resistant HT (Lovic et al., 2014), to the best of our knowledge, the effectiveness of this approach has not been yet tested in models of IH. Thus, further investigation in this specific field would be welcome.

In line with the pioneer study performed by Fletcher et al. (1992c) that established that carotid body (CB) ablation eliminated the hypertension related to CIH, McBryde et al. (2013) have shown that CB deafferentation, through bilateral carotid sinus nerve denervation, promotes an effective and lasting AH response in SH rats and reduces the overactive sympathetic activity. They have also demonstrated that associated with renal denervation, carotid sinus nerve denervation remains effective and produces a cumulative response (McBryde et al., 2013). In line with these findings, they propose carotid sinus nerve denervation as an effective AH treatment in patients with sympathetically mediated diseases (McBryde et al., 2013).

More recently, Burchell et al. have reviewed the potential of a new device for the control of arterial HT (Burchell et al., 2014). The ROX coupler device creates an anastomosis between the iliac artery and vein, diverting a calibrated amount of arterial blood into the venous system, reducing vascular resistance and increasing arterial compliance (Burchell et al., 2014). This non-pharmacological approach seems to be a promising tool in the management of patients with resistant HT due to its ability to provide an immediate and sustained reduction in BP (Burchell et al., 2014). The safety and efficacy of the ROX coupler in the treatment of this type of HT is now being evaluated in a European multicenter randomized study (Burchell et al., 2014). Positive results in patients with drug-resistant HT leave open the possibility of the use of the ROX coupler device becoming a new strategy for the management of HT related to OSA.

## Conclusions and future perspectives in the management of HT related to OSA

There is consensus that HT related to OSA is gaining more relevance as an independent nosological condition that needs a systematic approach to identify the best therapeutic strategy. One major challenge is gaining an understanding of whether the blockade of the reflex pathways triggered by CB activation is sufficiently effective to control BP in itself in HT related to OSA. Eventually, other pathways directly stimulated by hypoxia at a cellular level should be explored in depth and manipulated to attain relevant clinical control of these patients.

Drugs that have proved to be useful in essential HT treatment should be tested promptly in studies specifically designed for secondary HT induced by CIH. On the other hand, given the particularities of HT related to OSA, the recourse to tailored treatments should be considered as a possibility. Furthermore, it also appears to be imperative to look for new AHDs able to reverse HT quickly and effectively in patients with OSA as BP control is still not achievable in a significant proportion of these patients.

The contribution of animal models to this approach is unquestionable in terms of avoiding the confounding risk factors for HT that tend to be present in OSA patients. In addition, drugs that have been used as pharmacological tools to understand pathophysological mechanisms should now be investigated regarding their efficacy in reverting HT induced by CIH.

## Author contributions

Lucilia N. Diogo and Emília C. Monteiro wrote the manuscript and approved the final version.

### Conflict of interest statement

The authors declare that the research was conducted in the absence of any commercial or financial relationships that could be construed as a potential conflict of interest.
